# Supercontinuum Generation in Soft Glass Photonic Crystal Fibers Developed Based on 3D Printed Preforms

**DOI:** 10.1002/advs.202511930

**Published:** 2025-10-07

**Authors:** Pawel Wienclaw, Przemyslaw Golebiewski, Grzegorz Stepniewski, Bartosz Paluba, Pawel Socha, Adam Filipkowski, Dariusz Pysz, Wenzhong Liu, Andrzej Burgs, Rafal Kasztelanic, Ryszard Buczynski

**Affiliations:** ^1^ Faculty of Physics University of Warsaw Pasteura 5 Warsaw 02–093 Poland; ^2^ Department of Photonic Materials Department of Fiber Optics Technology and Quantum Systems Lukasiewicz Research Network – Institute of Microelectronics and Photonics al. Lotników 32/46 Warsaw 02–668 Poland; ^3^ Sygnis S.A al. Grunwaldzka 472 Gdansk 80–309 Poland; ^4^ School of Artificial Intelligence and Automation China‐Poland Joint Laboratory on Measurement and Control Technology Huazhong University of Science and Technology Wuhan 430074 China

**Keywords:** additive manufacturing, optical fibers, photonic crystal fibers, supercontinuum generation, soft glass

## Abstract

The readiness level of 3D glass printing technology for optical fiber development is explored. A preform of an air‐glass photonic crystal fiber is printed with synthesized in‐house lead borate glass using a custom‐made 3D printer. The fiber is single‐moded at a wavelength of 1.5 µm and has flat anomalous dispersion in the infrared range above 1.5 µm with a zero‐dispersion wavelength at 1.7 µm. An octave spanning supercontinuum from 1.1 to 2.2 µm is obtained by pumping the 22 cm long section of the printed fiber in the anomalous dispersion regime with 100 fs pulses at 1560 nm wavelength using a standard off‐the‐shelf fiber femtosecond laser. Despite internal structural defects, the results indicate that early‐stage 3D glass printing can enable the development of preforms for nonlinear fibers dedicated to supercontinuum generation with spectral widths comparable to state‐of‐the‐art manually assembled PCFs, but still with considerably higher losses.

## Graphical abstract

This research investigates whether modern 3D glass printing can be used to develop advanced photonic crystal fibers suitable for supercontinuum generation. The team designs and fabricates a lead‐borate glass fiber with high nonlinearity, achieving an octave‐spanning spectrum in the mid‐infrared range. 3D glass printing represents a promising alternative to the conventional fabrication of complex optical fibers using the stack‐and‐draw method, which requires manual preform assembly.

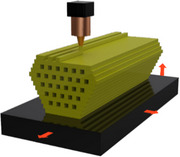

## 1 Introduction

The stack‐and‐draw technique is commonly used to fabricate glass‐based nonlinear photonic crystal fibers (PCFs) and various structured multimaterial fibers.^[^ ^1^, ^2^, ^3^ ^]^ With the photonic crystal fiber approach, all critical optical parameters of the fiber, such as dispersion characteristics, effective mode area, and nonlinear coefficient, can be controlled and optimized. However, this method requires the manual assembly of fiber preforms, which limits the scalability and robustness of fiber development.

The extrusion process has also been successfully reported for the development of preforms for highly nonlinear photonic crystal fibers. However, this method can only fabricate relatively simple fiber structures with a limited number of air holes and limited accuracy.^[^ ^4^, ^5^ ^]^ To overcome current limitations, the performance development should be automated. We can consider robotic assembly of the preforms based on rods and capillaries or 3D glass printing. Robotic assembly has been considered for several years; however, it has never been successfully implemented.^[^ ^6^ ^]^

The 3D printing approach is more successful, and this method can be considered the most promising fabrication technique for structured optical fibers made of glass.^[^ ^7^, ^8^, ^9^ ^]^ 3D printing of very complex fiber‐like structures with photocured polymer using two‐photon polymerization has been developed during the last decade.^[^ ^10^ ^]^ However, only very short sections of the fiber (a fraction of a millimeter) can be printed with this method. Moreover, the practical applicability and durability of these structures remain limited due to the use of polymer materials.

Since 2019, several different approaches have been considered for the 3D printing of silica glass and soft glasses.^[^ ^9^ ^]^ In the case of silica fibers, various methods based on 3D printing from organic inks with silica powder are classified as Direct Ink Writing (DIW) and Stereolithography (SLA).^[^ ^11^, ^12^, ^13^ ^]^ These methods require two‐step postprocessing of the printed preform structure, which consists of preform debinding to remove organic compounds and sintering to obtain pure silica glass preforms. These processes cause preform shrinkage and can induce additional deformation of preforms.^[^ ^14^, ^15^ ^]^ Therefore, this class of methods is well‐suited for the development of fibers with a simple core‐clad structure^.^^[^ ^16^ ^]^ However, Yazici et al. have recently demonstrated, for the first time, the successful development of a complex air‐glass microstructured preform for silica glass optical fibers using photocurable nanocomposites.^[^ ^17^ ^]^ These early, yet extremely important, results indicate a possible paradigm shift in the fabrication of air‐glass fiber structures using sintering processes within the technological chain of fiber development. We note, however, that the development of a complex all‐solid structure for glass‐based fibers using the SLA method still remains an unsolved issue. Similar limitations occur with methods for performing development based on laser‐assisted sintering (selective laser sintering, SLS)^[^ ^18^ ^]^ or direct powder melting, usually referred to as laser powder deposition methods LPD).^[^ ^19^, ^20^ ^]^

An alternative approach is based on direct glass printing from glass filament or bulk glass, usually referred to as fused deposition modelling (FDM).^[^ ^21^, ^22^ ^]^ The advantage of this method is that the printing process is performed with pure glass without the presence of any organic compounds. Therefore, a lower attenuation of fibers might be expected, since there is no need to remove unwanted compounds from the glass. In practice, this method is typically limited to soft glasses only, as it requires 3D printing at temperatures above their softening point. Several successful approaches for printing air‐glass structured fibers have been reported using oxide‐based glasses^[^ ^23^, ^24^ ^]^ and chalcogenide.^[^ ^22^, ^25^ ^]^ Recently, the first approach to fluoride glass fiber printing was reported by Galdo et al.^[^ ^26^ ^]^

Vertical and horizontal printing are considered for the FDM method. Vertical printing has limitations related to the length of the preform and single‐line printing due to air trapping between the printed glass lines. Therefore, vertical preform printing is well‐suited to air‐glass structures with a large fraction of air (suspended core fibers, antiresonant fibers). Horizontal printing faces challenges related to the collapse and deformation of air holes during preform printing.^[^ ^24^ ^]^ This method is better suited to printing photonic crystal fiber structures with a low relative hole size.

In this paper, we report, for the first time, the successful generation of a supercontinuum spanning over one octave in a highly nonlinear photonic crystal fiber fabricated from a horizontal 3D‐printed preform.

## 2 Development of Photonic Crystal Fiber Based on 3D Printed Preform

### 2.1 Synthesis and Characteristics of Highly Nonlinear Glass for 3D Printing Preforms

In the scope of previous studies,^[^ ^24^ ^]^ we designed and synthesized a SiO_2_‐stabilized lead borate glass, designated as CD‐16. The primary objective was to develop a thermally stable, highly nonlinear heavy metal oxide glass suitable for manufacturing microstructured fibers for supercontinuum generation. The glass composition was optimized to retain its amorphous state during prolonged thermal treatment in the 3D printing process, while maintaining a low viscosity to facilitate extrusion from low‐diameter nozzles. The chemical composition of the CD‐16 glass is outlined in **Table** **1**.

**Table 1:** The CD‐16 glass composition.

	% wt.	% mol
SiO_2_	5	11.88
PbO	71	45.42
CdO	6	6.67
ZnO	3	5.26
B_2_O_3_	15	30.77

To synthesize the glass, all substrates were carefully weighed according to the established chemical composition and mixed in an alumina mortar to form a homogeneous powder mixture. An additional 0.25 wt.% of Sb_2_O_3_ was added as a clarifying agent to the batch mass to avoid the presence of small air bubbles in the final product. The mixture was then placed in a platinum crucible and melted in a resistance furnace at 950 °C for 4 h in an air atmosphere. During the melting process, the glass was stirred using a silica glass rod and bubbled with purified, dry air (with a moisture content below 1 ppm). After melting, the glass was poured into a preheated graphite mould and annealed from 390 °C to ambient temperature at a cooling rate of 0.5 °C min^−1^. To design the 3D printing process, the thermal properties of CD‐16 glass were determined (**Table** **2**).

**Table 2:** Thermal properties of CD‐16 glass.

Thermal expansion coefficient (CTE) α [10^−7^ K^−1^]	93.2 [20–250 °C]
Dilatometry‐determined transformation temperature *T*_g‐dil_ [°C] log*η* = 13.4	**375**
DTA‐determined transformation temperature *T*_g‐DSC_ [°C]	**379**
Dilatometric softening point *T*_DSP_ [°C] log*η* = 11	**401**
Characteristic temperatures registered in Leitz heating microscope T [°C]	
Sphere temperature *T*_sph_ [°C] log*η* = 6	430
Hemisphere temperature *T*_hs_ [°C] log*η* = 4	480
Flow temperature *T*_flow_ [°C] log*η* = 2	558

**Figure 1:**
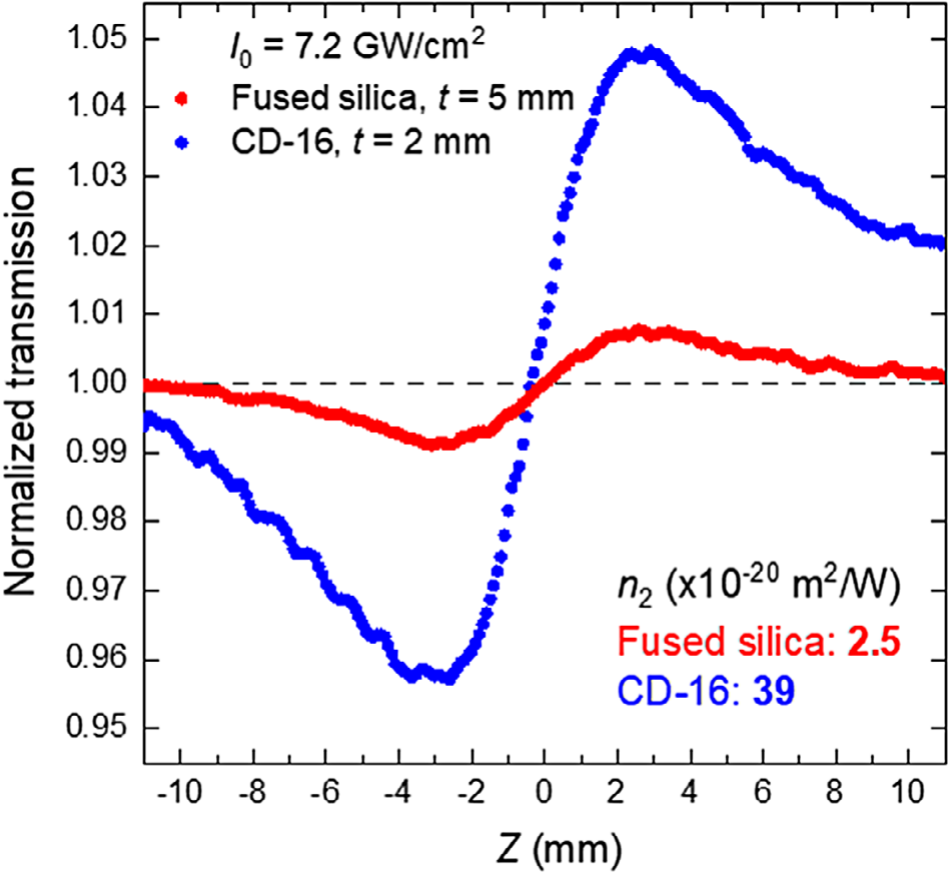
Closed aperture z‐scan traces recorded for fused silica glass (*t* = 5 mm) and CD‐16 glass, in‐house developed glass (*t* = 2 mm) for the same laser intensity in the focus.

We measured the nonlinear properties of the developed glasses using the z‐scan method.^[^ ^27^ ^]^ This commonly used technique enables straightforward determination of key parameters, such as nonlinear refraction and absorption. In our setup we used a femtosecond fiber laser (Fluence Halite 2) emitting 206 fs pulses at a wavelength of 1032 nm with 20 MHz repetition rate. The high quality beam was focused using a 75 mm focal length lens on the bulk glass sample placed on a motorized linear stage (25 mm travel distance). Moving the sample along the optical axis (z‐axis) around the focal point with a range longer than the Rayleigh range (*z*_R_ = 2.62 mm) enables observation of the self‐focusing phenomenon, which manifests as changes in the laser beam size far away from the focus. The measurement of paraxial laser beam intensity after the glass sample, using an aperture in front of the detector, allows us to determine the nonlinear index of refraction *n*_2_ from the empirical formula^[^ ^27^ ^]^:

(1) $$n_{2}\approx \frac{\sqrt{2}\lambda \Delta T_{p-v}}{2\pi I_{0}L_{eff}0.406{\left(1-S\right)}^{0.25}}\approx 0.554\frac{\lambda \Delta T_{p-v}}{I_{0}L_{eff}{\left(1-S\right)}^{0.25}}$$

where the normalized peak‐to‐valley transmittance Δ*T*_p‐v_ is obtained from the z‐scan trace recorded in the closed aperture configuration (**Figure** **1**). The aperture transmittance parameter *S* was set below 10%, which means that *S* < 0.1. The crucial parameter required for accurate *n*_2_ calculation is the light intensity *I*_0_ at the focal point. Therefore, the laser pulse temporal characteristic, beam shape, and size of the beam at the focus ω_0_ have to be well defined in the experiment. Another important factor about the sample, which has to be taken into account, is both the linear and nonlinear absorption. The linear absorption coefficient α was obtained from the transmission measurement at a low laser power without the focusing lens and by subtracting the Fresnel reflection. The absorption parameter is accounted for in the effective sample thickness *L*_eff_ = (1‐*e* ^α^ *^t^*)/*α*, where *t* is the sample thickness. The nonlinear absorption, primarily caused by two‐photon absorption, is observed as a drop in transmittance in the z‐scan trace for the open aperture, where the intensity of the entire beam is measured. In the laser power regime used in our experiment, a nonlinear absorption was only slightly observed in CD‐16 glass, and it was included in the final normalized transmittance traces obtained in the closed aperture configuration. An example of two traces registered for 5 mm thick fused silica sample and 2 mm thick CD‐16 glass at the same laser intensity in the focal point *I*_0_ = 7.2 GW cm^−2^ is shown in Figure 1.

To verify the correctness of the z‐scan measurements, we have measured as a reference the nonlinear refractive index of pure silica glass and other heavy metal oxide glasses (**Table** **3**). The obtained results are in very good agreement with previously reported results.^[^ ^28^, ^29^ ^]^ The nonlinear refractive index of the CD‐16 glass used to print the fiber preform was determined as *n*_2_ = 39×10^−20^ m^2^ W^−1^. This value is one order of magnitude higher than for silica glass^[^ ^28^ ^]^ and similar to other previously reported lead‐bismuth‐gallate soft glasses, namely PBG‐89 and CS‐1030.^[^ ^29^ ^]^ We note that the high nonlinearity of lead borate glasses is related to their composition and the presence of heavy metal oxides, which also influence their transmission properties. The attenuation of CD16 glass is 8 dB/m in the transmission window of 0.6–2.5 µm.^[^ ^24^ ^]^ This value is typical for heavy metal oxide glass.^[^ ^30^ ^]^ Therefore, the effective use of CD16‐based fibers is limited to specific applications, such as supercontinuum generation or other nonlinear phenomena, where a very short section of fiber (a fraction of a meter) can be utilized.

**Table 3:** Summary of the measured nonlinear index of refraction.

Sample	Thickness	*n*_2_ [x10^−20^ m^2^ W^−1^]	Power range [mW]	Reference measurements
Fused silica	5 mm	2.5 (2.41–2.56)	1570–1970	2.56 (1.9–2.8)^[^ ^28^ ^]^
PBG‐89	2 mm	30 (27–32)	600–1380	31^[^ ^29^ ^]^
CS‐1030	0.875 mm	42 (39–44)	600–1180	41^[^ ^29^ ^]^
CD‐16	2 mm	39 (38–39)	600–1380	this work

**Figure 2:**
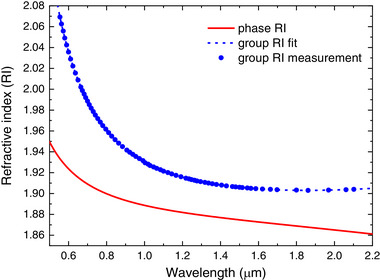
Measurements of phase and group refractive index of CD‐16 glass.

The dispersion of phase and group refractive index was measured in a broad spectral range from 0.5 µm to 2.2 µm using a Michelson interferometer configured for operating with incoherent white light. Both dispersion characteristics for CD‐16 glass are presented in **Figure** **2**. The Sellmeier coefficients were calculated based on the experimental results (**Table** **4**).

**Table 4:** The Sellmeier coefficients of CD‐16 glass.

B1	B2	B3	C1	C2	C3
2.2	0.317459	1.608194	0.017625	0.068473	122.5

### 2.2 Design of PCF Based on CD‐16 Glass for Supercontinuum Generation

We designed the PCF structure optimized for broadband supercontinuum generation in the femtosecond regime, pumped with commercially available laser sources emitting at a wavelength of 1560 nm. The key parameters in this case are single‐mode performance in the vicinity of the pump source, flat anomalous dispersion characteristics, and a Zero Dispersion Wavelength (ZDW) close to the pump wavelength.

**Figure 3:**
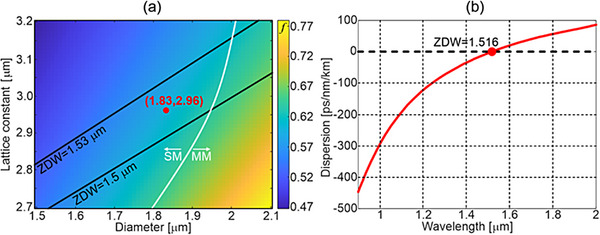
Dispersion characteristics and ZDW for a PCF based on CD‐16 glass optimized for supercontinuum generation: a) The color bar shows the value of the relative hole size parameter *f* for different geometrical parameters of photonic crystal fibers. The black lines mark regions of fiber geometrical parameters corresponding to the optimum position of the ZDW and indicate constant ZDW values (1.53 µm and 1.50 µm). The white line separates the parameter space into regions of single‐mode and multimode fiber performance calculated for a predicted pump wavelength of 1560 nm; b) The chromatic dispersion of the optimized PCF with a diameter of air holes d = 1.83 µm and the lattice constant Λ = 2.96 µm.

To effectively generate a supercontinuum signal, the ZDW should be close to the pump laser wavelength, preferably shifted toward shorter wavelengths to ensure pumping within the anomalous dispersion range.^[^ ^31^ ^]^ Therefore, 1500–1530 nm was selected as an optimum ZDW range. Secondly, the fiber should operate in the single‐mode regime, which depends on the relative hole size *f* defined as the ratio between the diameter *d* of air holes in the cladding and the lattice constant *Λ*, *f = d/Λ*. Photonic crystal fibers have unique features among all types of fibers. Namely, they can be endlessly single‐mode when the relative hole size is less than 0.43.^[^ ^32^ ^]^ Nevertheless, the PCF with the relative hole size *f*<0.43 requires a large number of air hole rings in the photonic cladding to mitigate confinement losses. Since we plan to use an early‐stage 3D glass printer for the preform development, we should limit the number of air holes in our design to reduce the complexity of the PCF structure. Therefore, we performed preliminary modeling to determine the parameters of the PCF made of CD‐16 glass, which guides effectively only the fundamental mode above a wavelength of 1.35 µm and has relatively low confinement losses in the proximity of the planned pump wavelength of 1.56 µm. We found that a PCF with a relative hole size below 0.63 and 3 rings of air holes in the photonic cladding is a fair trade‐off between single‐mode performance and low confinement losses (**Figure** **3**a).

Next, we performed a set of simulations to determine the optimum lattice constant and air hole diameters in the hexagonal lattice photonic cladding for the PCF with single‐mode performance and ZDW in the predicted range (Figure 3). Since 3D printing of glass fiber preforms is in an early stage, we can expect deviation of final fiber parameters relative to the designed values. Therefore, in our modeling, we focused not only on calculating optimum values but also on determining the parameter tolerance range that takes fabrication errors into account.

The variation of the lattice constant *Λ* and air hole diameter *d* is shown for the expected relative hole size and ZDW parameter (Figure 3a). The boundary between the single‐mode and multimode operating regimes is indicated by the white line. The area lying to the left of this curve and between the black lines representing the target range of the ZDW determines the preferred geometrical properties of the PCF. The tolerance range of the lattice constant is *Λ* = 2.90–3.00 µm, while the diameter of air holes is *d* = 1.75–1.87 µm. The optimum geometrical parameters for the PCF are: an air hole diameter of *d* = 1.83 µm and a lattice constant of *Λ* = 2.96 µm. The ZDW is 1516 nm for this fiber. The chromatic dispersion for the optimized fiber is shown in Figure 3b. We note that the proposed fiber is not designed as an endlessly single‐mode fiber, but it is optimized for single‐mode performance above a wavelength of 1.35 µm. In this case, we can utilize larger air holes and minimize confinement losses by employing a limited number of rings.

### 2.3 3D Printing of Photonic Crystal Fiber Preform

**Figure 4:**
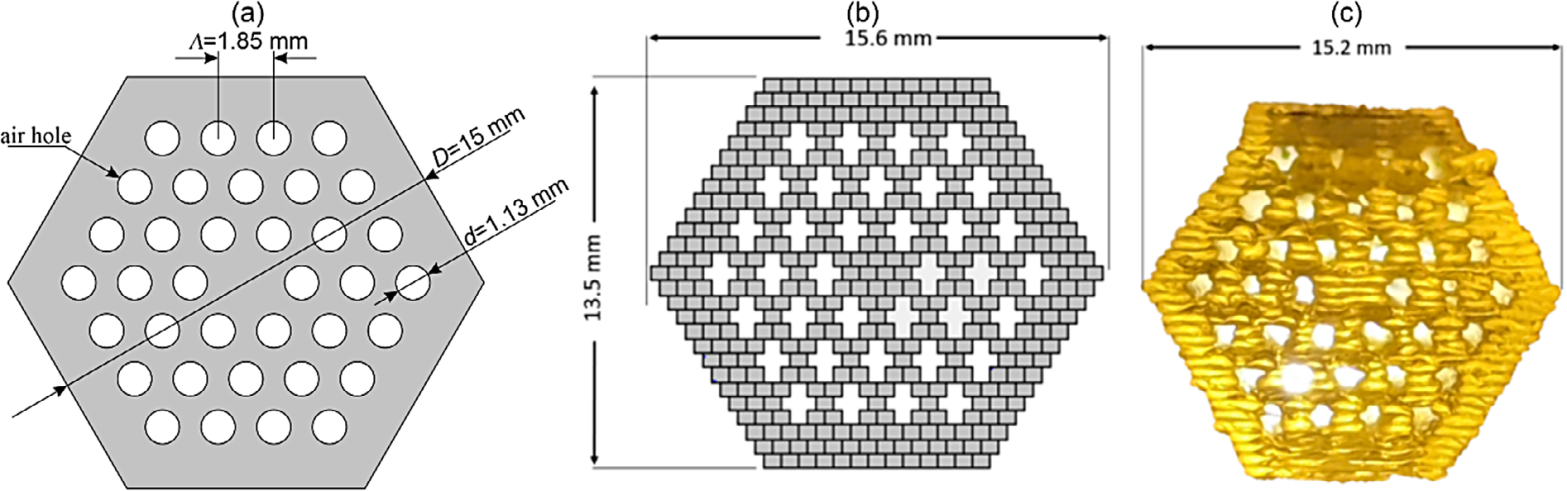
Fabrication of photonic crystal cladding a) a design of the PCF preform; b) CAD model of the cross‐section dedicated to horizontal 3D‐printing of photonic crystal cladding structure preform with the fiber core with a printing line of a height *h* = 0.5 mm, in vertical direction, and a width *w* = 0.6 mm, in horizontal direction; c) the glass 3D‐printed preform of the photonic crystal cladding.

**Figure 5:**
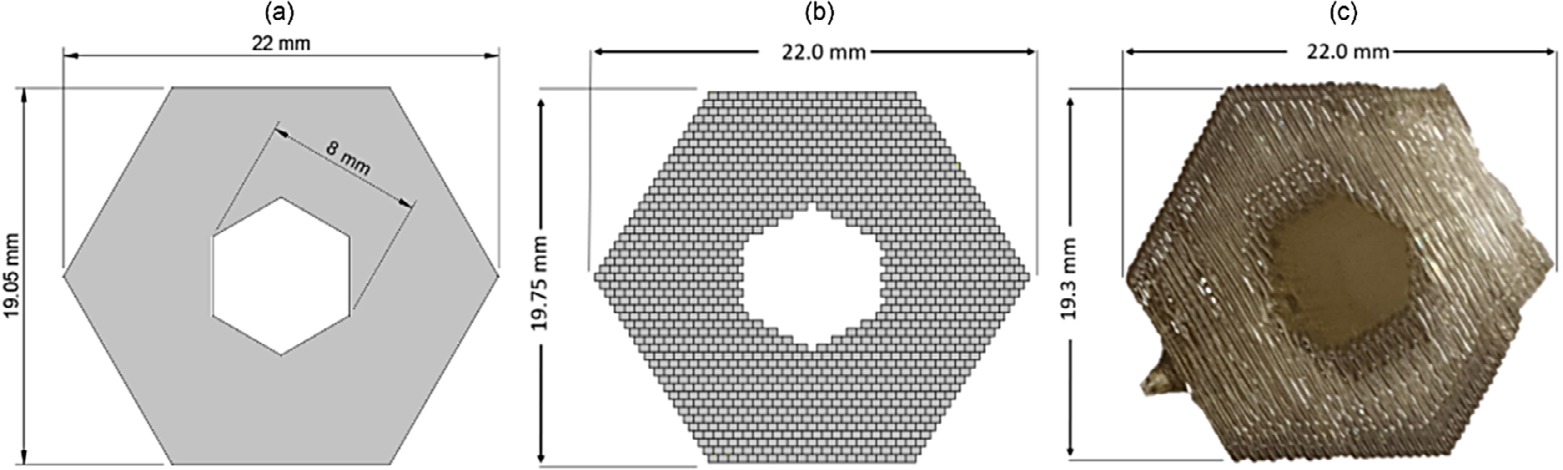
a) A general model of the external cladding tube; b) CAD model of the cross‐section dedicated to horizontal 3D‐printing with a printing line of a height *h* = 0.42 mm in vertical direction, and a width *w* = 0.5 mm in horizontal direction; c) the glass 3D‐printed external cladding tube.

The PCF preform (**Figure** **4**a) is composed of two independently printed components: the photonic crystal fiber cladding with core (Figure 4) and the external cladding tube (**Figure** **5**). Fabrication was performed using a dedicated 3D glass printing system, previously described in detail by Golebiewski et al.^[^ ^24^ ^]^ The preform was printed horizontally, line by line, to mimic the stack‐and‐draw assembly method with individual rods.

**Figure 6:**
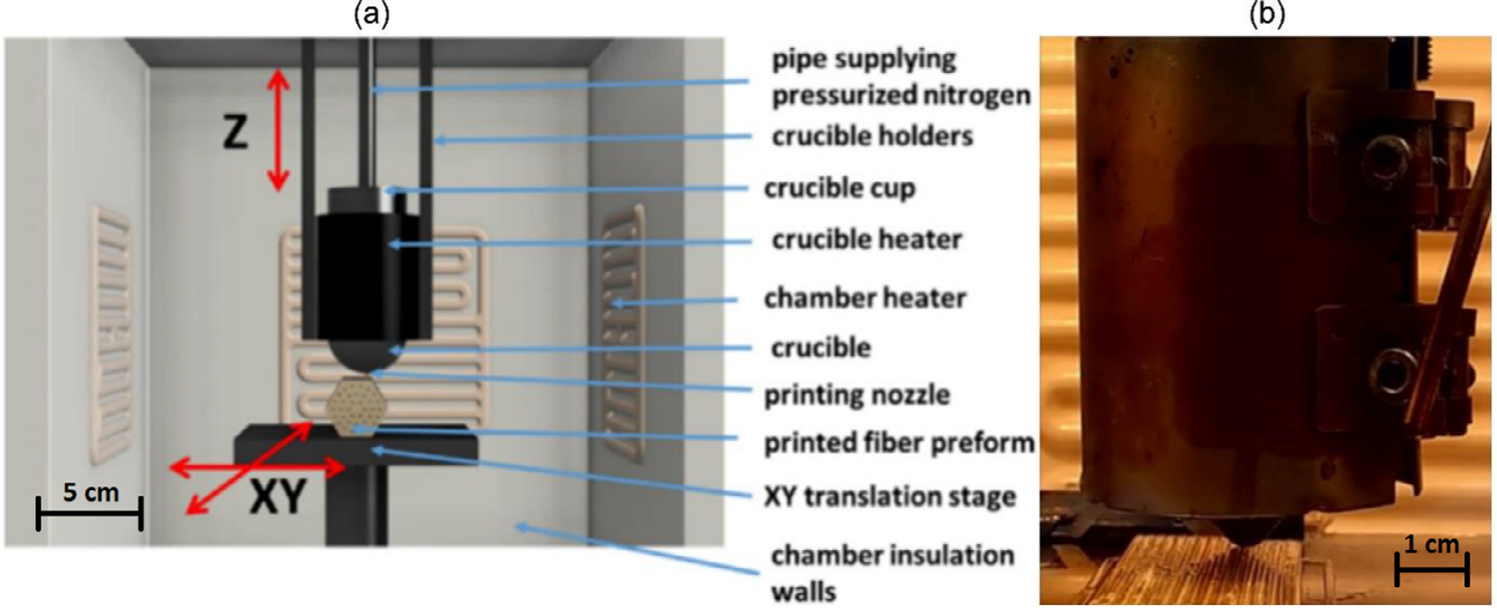
3D glass printer for automated fabrication of PCF preforms: a) The schematic of the horizontal printing of the PCF preforms: the printer head with crucible allows vertical positioning, translation stage ensures horizontal movement, b) the printer head during the fabrication of the glass preform.

The printer head with crucible allows movement in the vertical direction Z, while horizontal movement is provided by the printing bed mounted on a precision XY translation stage (**Figure** **6**b). Glass extrusion was achieved using a pneumatic pressure pump system through a stainless steel cylindrical crucible equipped with an interchangeable cone‐shaped nozzle with a diameter of 0.8 mm, operating within a thermally controlled chamber (30 × 30 × 40 cm^3^), as shown in Figure 6. The cylindrical crucible has outer and inner diameters of 38 and 34 mm, respectively, and a height of 90 mm. It is dedicated to thermally process glass cylinders with an outer diameter of 30 mm and a height of 80 mm. This corresponds to 250 m of printed line in a single printing process. Our preliminary printing test showed that after printing, the individual lines do not retain a circular cross‐section but are slightly flattened. This results from the elevated temperature of the printing process, during which the glass becomes soft and malleable. As a consequence, adjacent printed lines fuse together, forming a coherent glass block. However, each printed line lacks a circular cross‐section, which must be taken into account when designing the optical fiber preform (Figures 4b and 5b). When using a circular nozzle with a diameter of 0.8 mm, the extruded filament forms a line that can be approximated by a rectangle rather than a square. Its height in the vertical direction is smaller than its width in the horizontal direction, primarily due to the flattening effect that occurs during deposition, which is influenced by nozzle pressure, surface adhesion, viscosity, and gravity.

To develop a preform of the designed PCF, the CD‐16 glass blocks were preheated to 530 °C, while the chamber and printing bed were maintained at 340 °C and 350 °C, respectively. The heating rate during the warm‐up phase was 1 °C/min, and the cooling rate was set to 0.5 °C/min. The horizontal printing orientation was selected to ensure uniform longitudinal thickness and preserve consistent cross‐sectional characteristics along the fiber length. The imposed temperature gradient between the extruded glass and the surrounding environment was carefully optimized:

Glass extrusion was initiated and sustained using a pneumatic system supplied with pure nitrogen at a pressure of 14 bar. The XY translation stage was operated at a constant speed of 5 mm/s to ensure dimensional consistency and surface quality of the printed structure.

The glass 3D printed preform of photonic crystal cladding with a core has a 15 mm diagonal and 70 mm length. The structure consists of three rings of air holes, each with a diameter of *d* = 1.1 mm, arranged in a hexagonal lattice with a constant *Λ* = 1.85 mm, yielding a relative hole size (*d*/*Λ*) of 0.61 (Figure 4c). The glass 3D printed cladding tube has 22 mm diagonal, an inner diameter of 7.5 mm, and a length of 79 mm (Figure 5c).

We note that elevated chamber temperatures resulted in non‐uniformity of air hole sizes in the photonic cladding preform (Figure 4c). Some of the air holes in the external ring merged due to the low surface tension effects in the softened glass. A similar error (merging neighbouring air holes) was reported previously by us for another subpreform with a similar design (three rings of air holes).^[^ ^24^ ^]^ We noticed that errors were non‐symmetrical and always occurred on the same side of the preform–the side closest to the heated translation XY stage (Figure 6a). The glass was overheated from this side, and due to the low surface tension effects in the softened glass (exposed to too high a temperature for a long time in the first printed layers), this resulted in deformation and the merging of several air holes into larger ones. We suppose that this is a technical issue related to overheating of the base XY translation plate. The 3D printer lacks an active monitoring and control system for heating the XY translation plate, which would otherwise reduce its temperature during the printing process. We expect that reducing its temperature during the printing of the upper layers would solve this problem. Other approaches to consider include printing additional glass layers at the bottom of the preform to isolate the first air holes from the base XY translation plate, increasing the number of air holes (by adding more rings), or reducing the relative size of the air holes during subpreform printing.

To summarize, we suppose that the temperature setting and control in the chamber and at the XY translation plate remain still not optimal during this preform printing. On the other hand, insufficient heating may lead to induced thermal stress and interfacial cracking after the preform has cooled down to room temperature. Therefore, although we successfully printed the PCF preform, we note that heat management is not optimized in this process and requires further adjustment.

### 2.4 Fiber Development and Assessment of its Parameters

The fiber drawing process was separated into two distinct steps. The drawing process was performed using a fiber drawing tower dedicated to soft glass processing, equipped with a furnace capable of reaching a maximum temperature of 1000 °C.

In the first fiber drawing process, every preform (the external cladding tube and the photonic cladding preform) was drawn separately on the drawing tower to form two subpreforms. The aim of this process was to close air holes between printed lines and obtain a solid glass structure with designed air holes. Additionally, the process reduced the preform diameters to ensure a good fit between the two components of the preform.

**Figure 7:**
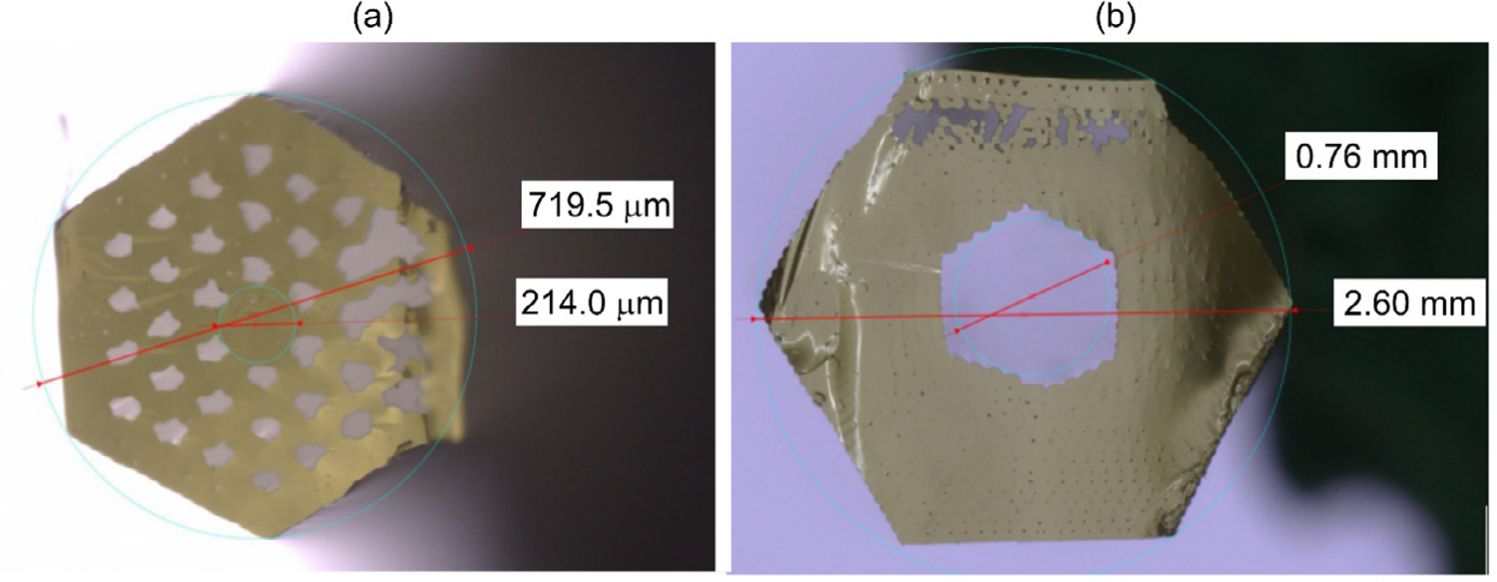
The subpreforms of the PCF: the subpreform of the photonic cladding a) and the subpreform of the external hexagonal cladding b).

The photonic crystal cladding preform was drawn into a subpreform with a diagonal of 0.72 mm and a length of 300 mm (**Figure** **7**a). The hexagonal external cladding preform was scaled down to a capillary with an outer diameter of 2.6 mm and an inner diameter matching the diagonal of the photonic cladding subpreform (Figure 7b).

The next step involved placing the PCF subpreform inside the capillary, and both were drawn together to form the final optical fiber. During this process, the self‐pressurization effect was utilized to maintain the air holes in the fiber structure without the need for external pressure. Simultaneously, the air pressure was reduced in the area between the two subpreforms to ensure good glass integration. The fiber drawing parameters (processing temperature, drawing speed, and feeding speed) were optimized to reduce the relative hole size in the photonic cladding and to obtain final geometrical parameters of the fiber close to the designed values. All parameters of the drawing processes are detailed in **Table** **5**.

**Table 5:** Parameters of the fiber drawing processes.

Glass component drawing/Parameters	photonic cladding subpreform	External cladding subpreform	Fiber
Temperature [°C]	525	512	652
Feeding speed [mm min^−1^]	0.1	0.18	0.09
Drawing speed [m min^−1^]	1.05	0.59	4.2

**Figure 8:**
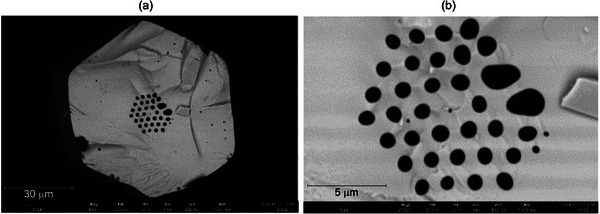
SEM images of the drawn fiber: cross‐section of the fiber a) and the photonic cladding b).

The final fiber has a hexagonal shape with an external diagonal of *D* = 80 µm, while the fiber core diameter is *d*_c_ = 3.8 µm (**Figure** **8**). The diagonal of the photonic crystal cladding is *D*_c_ = 19 µm, with the lattice constant *Λ* = 2.9 µm, and an average cladding air‐hole diameter of *d* = 1.8 µm (the relative hole size *f* = 0.62). Overall, the developed structure matches the designed one, but defects in the external ring of the photonic crystal cladding emerged during subpreform processing. These defects included the merging of air holes from the outer ring and some residual pores in the external solid cladding area of the fiber. These defects originated in the first fiber drawing process and were carried over to subsequent steps; they resulted from errors during the preform 3D printing process. Specifically, merging of air holes in the photonic cladding occurred when air holes were not fully separated along the printed lines of the subpreforms. The residual pores in the cladding area are formed from small air bubbles trapped within the glass structure during 3D printing.

All these defects could not be corrected at later stages of the fiber development process, and the application of higher temperatures during drawing only enlarged them (the internal pressure of trapped gas increases).

**Figure 9:**
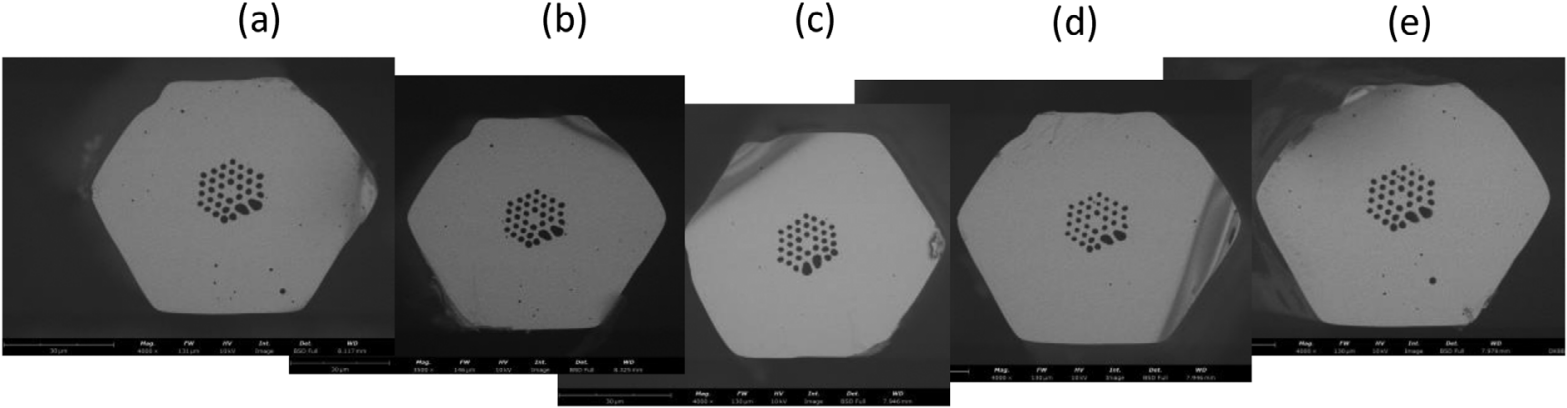
SEM images of the drawn fiber at different lengths of 0 m a), 2 m b), 4 m c), 8 m d), and 11 m e).

The uniformity of the fiber internal structures was verified by taking multiple images. We observed the presence of small air holes in various places of the fiber, while the size and shape of the core and air holes forming photonic cladding remain the same over a distance of 11 m (**Figure** **9**). Small residual pores were a result of air bubbles trapped in the fiber preform during 3D printing, as explained above.

## 3 Measurements of Linear Properties of the Fiber

**Figure 10:**
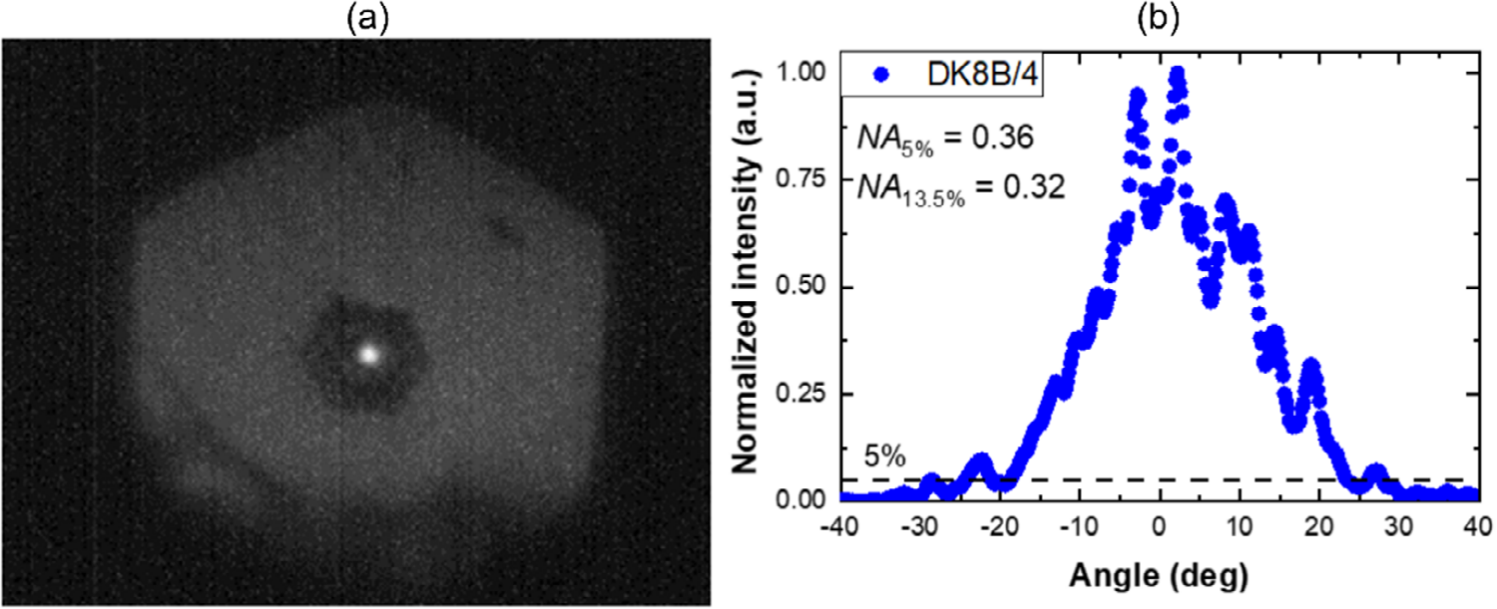
Single‐mode operation in the fiber core at a wavelength of 1565 nm a), Angular intensity distribution at the far field recorded for the wavelength of 1565 nm b).

We experimentally verified, using a near‐infrared InGaAs camera, that the laser beam is well confined in the core, despite the deformation of the photonic crystal cladding. The registered output beam indicates that the fiber is a single mode at a wavelength of 1565 nm (**Figure** **10**a). Next, we measured the numerical aperture (*NA*) of the fiber to confirm the single‐mode performance of the fiber. For this purpose, the angular intensity distribution in the optical far field was measured by exciting the fiber core at a wavelength of 1565 nm using a high *NA* microscope objective (x40/0.65) (Figure 10b). Taking into account the 5% criterion of intensity drop, the numerical aperture of the fiber is *NA* = 0.36. Assuming the Gaussian approximation of the beam from a single‐mode fiber, the mode field diameter equals *MFD* = 3.0 µm, while the geometrical core size is 3.8 µm. Based on measured MFD and nonlinear refractive index *n*_2_ of CD‐16 glass, the nonlinear coefficient γ can be calculated with the equation *γ* = (2*π* × *n*_2_)/(*λ* × *A*_eff_), where *A*_eff_ denotes the effective mode area. The nonlinear coefficient *γ* for the considered fiber is 222 (W*km)^−1^ for 1560 nm wavelength.

**Figure 11:**
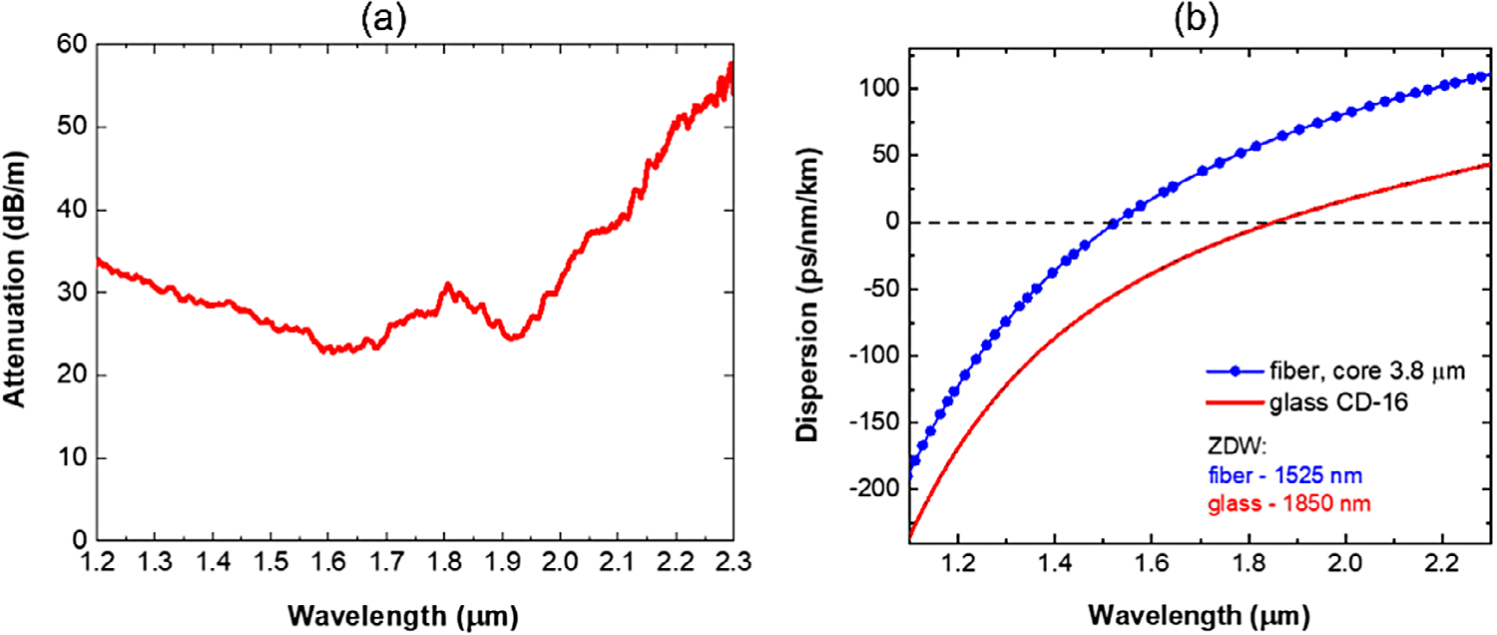
The measured attenuation of the fabricated nonlinear PCF made of CD‐16 lead borate glass a) and dispersion characteristics of the fiber and material dispersion of bulk glass CD‐16 b).

Next, the loss measurement was performed using the cut‐back method. As the light source, the supercontinuum laser (Samba, Leukos) was used, which was subsequently coupled to the fiber core with an aspheric lens (*f* = 6.24 mm, *NA* = 0.4). The output spectra in the range of 1.2–2.3 µm were recorded using the optical spectrum analyzer (Yokogawa, AQ6375B). A relatively high attenuation of 25–30 dB m^−1^ was measured in the range 1.3–2.0 µm (**Figure** **11**a). Attenuation of bulk CD‐16 glass after casting is 8 dB m^−1^.^[^ ^24^ ^]^ The strong increase of fiber loss above 2000 nm results from the rise in CD‐16 glass absorption in this range. The main reason for high attenuation is the presence of deformed air holes in the photonic cladding. Nevertheless, due to the high nonlinearity of the glass, only a very short fiber section is required to generate a supercontinuum. Therefore, a high attenuation of the fiber is not a critical issue.

Dispersion characteristics of the fiber were measured using the white light interferometric method with the Mach‐Zehnder interferometer in a broad spectral range 1.0–2.3 µm (Figure 11b).^[^ ^33^ ^]^ The dispersion characteristic is in very good agreement with the designed one (Figure 3b) despite deformation and diameter non‐uniformities of air holes in the photonic cladding. The zero dispersion wavelength (ZDW) occurs at 1525 nm, indicating that pumping in the anomalous dispersion regime is considered if a femtosecond erbium fiber laser is used. The dispersion characteristic is flat in the infrared range, varying between −75 and 68 ps nm^−1^ km^−1^ for the broadband infrared wavelength range of 1.3–1.9 µm. This type of dispersion characteristics favors a broadband supercontinuum generation driven by soliton fission.^[^ ^34^ ^]^

## 4 Supercontinuum Generation

### 4.1 Experimental Results

For supercontinuum generation in the printed highly nonlinear PCF, we used the femtosecond fiber laser operating at a wavelength of 1560 nm (Menlo Systems C‐780). This laser emits femtosecond pulses with a duration of *t*_p_ = 100 fs, with a high repetition rate of *f*_rep_ = 100 MHz and a maximum average power of *P_avg_* = 500 mW. Therefore, the peak power and energy of the pulse, which are *P*p = 50 kW and *E* = 5 nJ, can be obtained, respectively.

**Figure 12:**
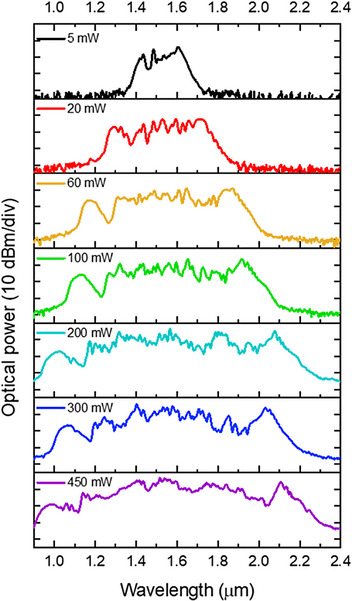
The measurement of supercontinuum generation in a 21 cm long fiber section pumped with a femtosecond laser with various average powers.

To couple the light into the fiber core, an aspheric lens with a focal length of *f* = 4.5 mm and a numerical aperture of *NA* = 0.55 was used. It provides a fiber coupling efficiency of 20%. The output spectrum was delivered via single‐mode fiber SM‐2000 to the near infrared spectrometer with the sensing range 0.9–2.5 µm and spectral resolution of 4 nm (NIRQuest, Ocean Optics). For the experiments, we used a 21 cm‐long section of the fiber. The spectra of SC generation for various laser powers are presented in **Figure** **12**.

The spectral broadening of the pulse can be observed at an average laser power of 3 mW, corresponding to 6 pJ of energy and a peak power of 60 W coupled to the fiber core (coupling efficiency ≈ 20%). Due to the very high nonlinear refractive index of the glass (*n*_2_ = 39×10^−20^ m^2^ W^−1^) and small core size, which results in a small effective mode area *A*_eff_ = 7.06 µm^2^, this fiber is characterized by a high nonlinear coefficient *γ* = 222 (W*km)^−1^ at the pump wavelength of 1560 nm. At the maximum laser power (450 mW), measured behind the coupling lens, a supercontinuum spectrum spans from 940 to 2230 nm, considering the −20 dB dynamic range, which gives a bandwidth of 1290 nm, indicating more than one octave of spectrum broadening. The maximum output power, measured with a thermal sensor, did not exceed 100 mW. The characteristic wings on both the short and long wavelength sides of the SC spectrum, which shift outward with power increase, suggest the presence of a red‐shifting soliton and a blue‐shifting dispersive wave as the main broadening mechanisms in this fiber, pumped in the anomalous dispersion range, typical for soliton fission‐driven supercontinuum generation using femtosecond lasers.^[^ ^35^ ^]^

### 4.2 Modeling of Generated Supercontinuum

We have performed numerical simulations of the supercontinuum generation in the considered setup. To solve the Generalized Nonlinear Schrodinger Equation for the electromagnetic field propagating in the optical fiber of a given set of physical characteristics, we have used the split‐step method commonly used for this purpose.^[^ ^34^, ^36^ ^]^

**Figure 13:**
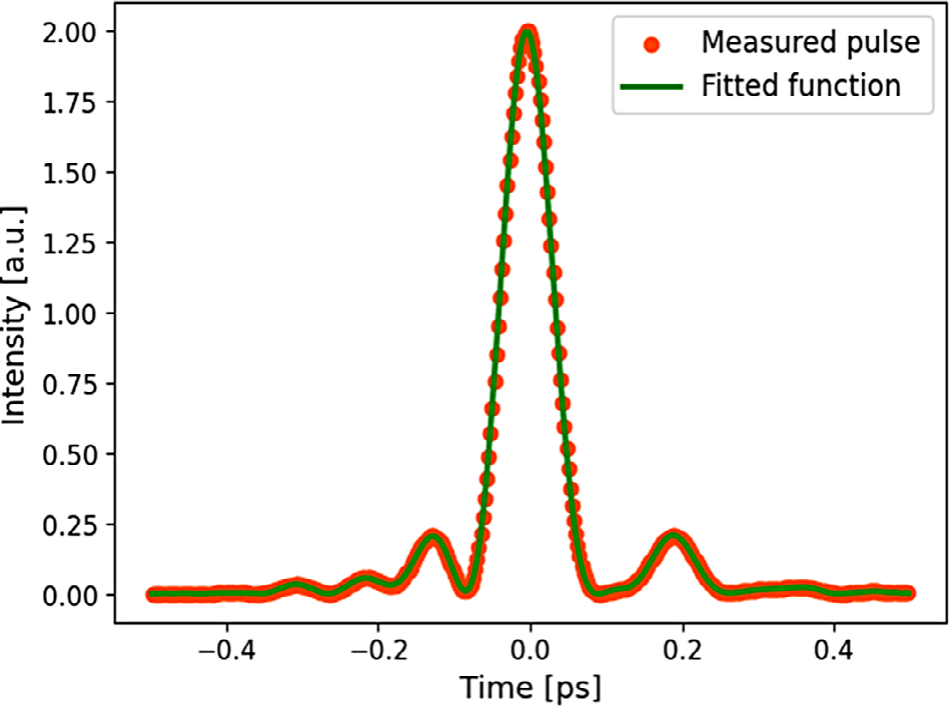
Frequency‐Resolved Optical Gating (FROG) measurements of a pump pulse shape(Menlo C‐780 laser, 100 fs pulse length with central wavelength of 1560 nm) and a polynomial fitting.

To conduct modeling matching experimental conditions, we retrieved the exact shape of the laser pulse used for supercontinuum generation. We used Frequency‐Resolved Optical Gating (FROG) measurements to determine the relation between the intensity distribution and time in the femtosecond pump pulse (**Figure** **13**). The Gaussian‐shaped part of the laser pump pulse accounts for 87% of the total peak power delivered by the laser. Only this fraction of the total peak power has an impact on the supercontinuum generation process.^[^ ^37^ ^]^

Anyway, we incorporated into our supercontinuum model the exact shape of the pulse (with a full width at half maximum ≈100 fs), its central wavelength of 1560 nm, and laser repetition rate of 100 MHz. Furthermore, the full measured optical characteristics of the fiber and glass material were also included, i.e., dispersion *D* and linear power attenuation α as functions of frequency, a nonlinear refractive index *n*_2_ = 3.9×10^−19^ m^2^ W^−1^ and a Raman response function *h*(*τ*) described by two parameters *τ*_1_ = 5.5 fs and *τ*_2_ = 32 fs with a coefficient *f*_R_ equal to 0.18.

**Figure 14:**
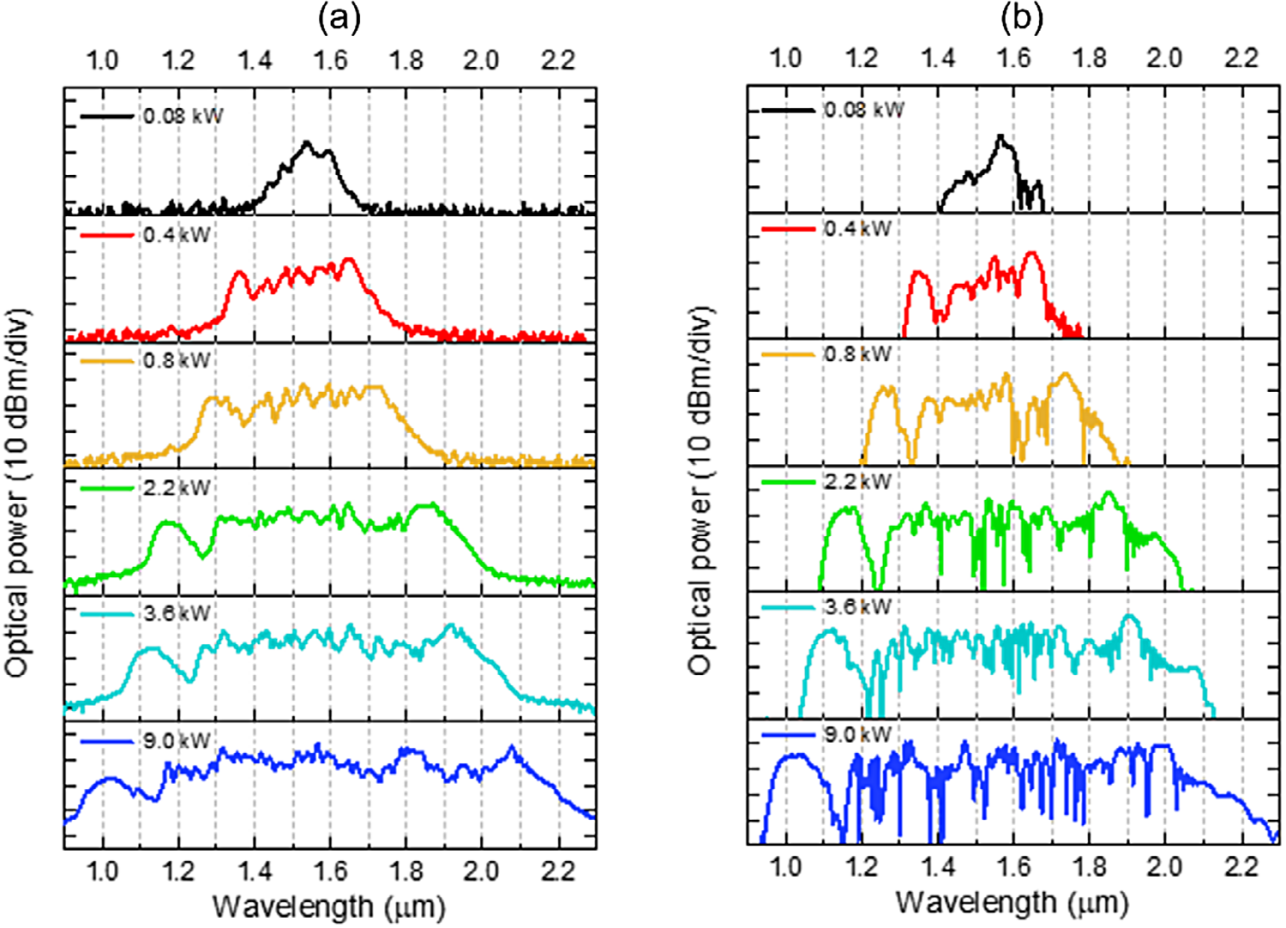
Comparison of supercontinuum spectra for selected peak powers of the input pulse. Measured results a), obtained numerically considering 20% coupling efficiency b).

Calculations were conducted assuming the coupling efficiency of laser pulses equal to 10 or 20% for a fiber length of 210 mm and for a wide range of average laser powers *P*_av_ from 0.2 mW up to 350 mW, which corresponds to experimental conditions. The selected set of calculated supercontinuum spectra, together with the peak powers of the pump pulses, is presented in **Figure** **14**.

**Figure 15:**
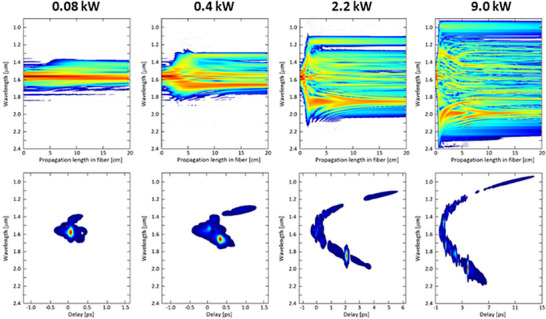
Modeling of pulse evolution during its propagation in 20 cm long section of nonlinear optical fiber (top images) with spectrograms (bottom images) for selected peak powers.

Comparing numerical results with experimental data for the same fiber length of 21 cm (Figure 14), we can notice a correct spectrum retrieval. Furthermore, for selected input powers, full maps of light intensity evolution along the fiber and spectrograms are plotted in **Figure** **15**. As one can predict, the significant broadening of the laser pulse is only observed when the power is high enough. The maximum width of the spectra is reached after a short propagation distance of ≈10–40 mm, depending on pulse power. For the remaining part of the fiber (40–210 mm), the shape of the spectra changes slightly, but their width remains constant. The mechanism of supercontinuum generation is typical for highly nonlinear fibers pumped with femtosecond lasers around the ZDW.^[^ ^34^ ^]^ Initially, we observe self‐phase modulation. Next, solitons are generated and shifted toward longer wavelengths along propagation distance, and finally, soliton fission occurs, and a dispersion wave is generated at a wavelength shorter than the initial pump pulse. Later, a set of cascaded nonlinear phenomena occurs, generating a broadband continuous spectrum.

**Figure 16:**
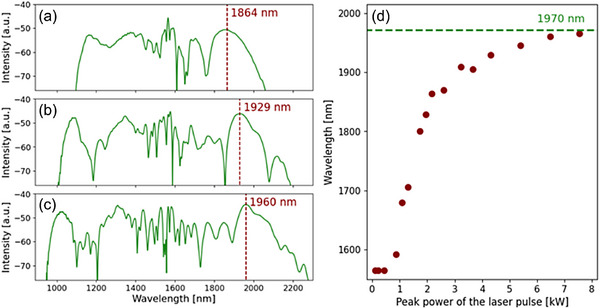
Simulated supercontinuum spectra obtained for 18 mm of propagation for peak powers of the laser pulse equal to: 2.2 kW a), 4.3 kW b), and 6.5 kW c) with marked solitons’ positions. The wavelength of the soliton maximum as a function of the source peak power d). The coupling efficiency was 20%.

We observed an evident relation between the soliton redshift and the peak powers, as well as the simultaneous formation of wide, smooth, blue‐shifted bands of high intensity–dispersive waves. Below a certain level, the supercontinuum generation is not observed, but starting from ca. *P*_av_ = 40 mW (corresponding to the peak power of 0.9 kW), solitons are clearly visible. Their positions are shifted toward longer wavelengths as they propagate in the fiber up to a certain maximum value. For different power levels, we chose a single fiber length of 18 mm, at which the soliton positions were fixed, and plotted the relationship between these positions and the peak powers of the laser. It emerges that the higher the input power is, the more red‐shifted the final position of the soliton is (**Figure** **16**a–c). Apparently, the soliton shift tends from below 1600 nm to a value of 1970 nm (Figure 16d).

## 5 Conclusion

The primary objective of the above research is to determine whether the current state of the art in 3D glass printing enables the development of highly nonlinear photonic crystal fibers suitable for supercontinuum generation.

For this purpose, we designed and developed a photonic crystal fiber with optical parameters optimized for anomalous supercontinuum generation in the femtosecond regime. The fabricated structure, based on a 3D printed preform PCF, has a core with a diameter of 3.7 µm and a hexagonal photonic cladding composed of three rings of air holes with a diameter of 1.8 µm and a lattice constant of 2.9 µm. We note that the fabricated fiber does not correspond to the design. Its internal structure has defects in the outer rings of the photonic cladding, resulting in high fiber attenuation (over 20 dB m^−1^). The shape and size of the air holes in the photonic cladding vary and differ from the designed ones. Nevertheless, the fabrication imperfections are small enough to have a very limited influence on the fiber dispersion characteristics, the core size, and other effective parameters of the fiber, which remain similar to the designed ones.

The fabricated fiber is single mode for a predicted pump wavelength of 1560 nm, with a numerical aperture of *NA* = 0.32, flat dispersion varying between −75 and 68 ps nm^−1^ km^−1^ for the broadband infrared wavelength range 1.3–1.9 µm and ZDW at 1525 nm, enabling to pump at anomalous dispersion at near zero‐disposition range.

Due to high nonlinearity of lead‐borate glass, with a nonlinear refractive index of 39×10^−20^ m^2^ W^−1^ the fabricated fiber has a very high nonlinear coefficient *γ* = 222 (W*km)^−1^ for 1560 nm wavelength taking into account the core diameter of 3.7 µm, suitable for efficient input pulse coupling (at least 20%) with the commercial femtosecond lasers. We note that our 3D printing of preforms is not suitable for silica glass yet (too low processing temperature offered by our system), and other soft glasses should be further verified for 3D printing, since the glass must withstand long‐term thermal exposure without crystallization.

Experimental results show that over one octave of supercontinuum generation spans from 940 to 2230 nm, considering a ‐20 dB dynamic range, which yields a bandwidth of 1290 nm using 100 fs pulses with an energy of 6 pJ. These results are similar to those previously obtained with PCFs made of soft glass with manual assembly of the preform.^[^ ^38^, ^39^ ^]^

The obtained results indicate that at this early stage, 3D printing of preforms presents an alternative method for developing highly nonlinear PCFs with comparable performance in terms of generated supercontinuum spectral width, albeit with significantly higher losses. The main advantages are reproducible, error‐free automated preform development, which eliminates the need for manual assembly, and a reduction in time and material consumption during the development of rods and capillaries required for the traditional stack‐and‐draw method.^[^ ^1^, ^2^ ^]^ Moreover, the printed preforms are not limited by their length as in the case of the stack and draw method. For manual assembly, preforms of PCFs are typically short (usually up to 25–100 cm long, depending on the number of rods on the diagonal of the preform), as rigid rods are required for manual assembly of the preforms. As a result, 3D preform printing can offer significant cost and time reductions in the future for the fabrication of soft‐glass PCFs, a reduction in human errors during preform assembly, while maintaining quality and supporting high‐volume production. This method can have a significant impact on the development of specialty fibers with optical parameters that standard fibers cannot provide (e.g., very high nonlinearities, optimized dispersion characteristics, complex multicore fibers, with artificially anisotropic glass, low‐latency hollow‐core PCFs, or free‐form nanostructured fibers).

In general, we can identify two strategies for fabricating air‐glass optical fibers with complex structures today: first, direct fiber development in a single step (“print‐to‐fiber”), and second, a two‐step process: “preform + fiber.”

The first approach can be based on direct 3D printing with submicron resolution of the fiber with dedicated photopolymers (STL techniques). However, if we focus on glass fiber, a UV‐curable or thermoplastic polymer binder with silica nanoparticles has to be used. Although a fiber structure can be printed with various processes (SLA, two‐photon polymerization), two additional processing steps are required to obtain a final fiber: removal of polymer binder and sintering to remove organic materials. Therefore, this “one‐step” process requires at least 3 separate processes before we can obtain the final fiber. Automatization of this process sequence is feasible, but not straightforward. Additionally, the length of printed fiber obtained in the single process is limited by the debinding process.

The second approach, by definition, consists of two processes: the printing of preforms (or, as in our case, printing subpreforms and their manual assembly), and fiber drawing at the fiber drawing tower. In this case, automation of preform development is feasible. However, fiber drawing requires additional work related to preform preparation before the drawing process, which is likely to be automated. The advantage of this method is that it allows for the production of several kilometers of identical fiber during a single process.

In the case of our considered horizontal 3D glass printing, several issues need to be addressed, including uniform heating of the preform and precise control of the complex heat transfer dynamics of molten glass during the lengthy printing process, which lasts several hours. The horizontal 3D printing of glass preform is only one of several recently considered additive manufacturing methods for future fiber development.

We note that in the present case, we reproduced a structure of photonic crystal fiber that could be developed using a standard stack and draw method. The horizontal 3D printing offers significantly more freedom in designing the preform structure to obtain more advanced optical properties (nonlinear fibers with two ZDWs), since the size and arrangement of air holes are not constrained by any symmetry requirements. To fully leverage these opportunities, a new design method should be further developed,^[^ ^40^ ^]^ and structured optical fibers composed of two glasses instead of air‐glass structures should be considered.^[^ ^41^ ^]^

## Conflict of Interest

The authors declare no conflict of interest.

## Author Contributions

R.B. and A.B. conceptualized the research project; R.B. and R.K. designed the experiments; P.W., P.G., G.S., R.K., W.L. and A.F. conducted the experiments; P.W. and A.B. developed and operated 3D glass printer; P.G. and P.S. synthesized glass, P.W. fabricated preforms, P.G., D.P. and A.F. fabricated final fibers; R.K. and R.B. designed the fiber, B.P. performed nonlinear simulations, P.W., P.G., P.S. and G.S. measured glass properties, P.W., P.G., and G.S. measured fiber properties, R.B., R.K. W.L. and A.B. analysed experimental results, P.W., P.G. B.P., A.F., P.S. and R.B. wrote the manuscript draft and prepared the figures; all coauthors contributed to revision and final version of the manuscript.

## Data Availability

The data that support the findings of this study are available from the corresponding author upon reasonable request.
